# Automated identification of MRI series using a hierarchical modular machine-learning pipeline

**DOI:** 10.1186/s41747-026-00740-z

**Published:** 2026-05-28

**Authors:** Mariusz J. Kujawa, Matías Fernández‑Patón, Leonor Cerdá Alberich, Diana Veiga-Canuto, Luis Martí‑Bonmatí

**Affiliations:** 1 Grupo de Investigación Biomédica en Imagen (GIBI230), Instituto de Investigación Sanitaria La Fe, Valencia, Spain; 2 2nd Department of Radiology, Medical University of Gdansk, Gdansk, Poland; 3 Área Clínica de Imagen Médica, Hospital Universitario y Politécnico La Fe, Valencia, Spain

**Keywords:** Artificial intelligence, DICOM, Magnetic resonance imaging, Multicentre study, Series classification

## Abstract

**Objective:**

The volume and diversity of large MR imaging datasets require efficient automated labelling tools for cataloguing MR series, as manual annotation is impractical and costly. However, relying on DICOM header fields alone is unreliable because sequence descriptors are heterogeneous and locally defined, frequently missing or incorrect, and may be altered or removed during anonymisation.

**Materials and methods:**

We developed an AI-based modular model to classify MR series. The pipeline comprises five sequential classifiers (Family, Weighting, Fat Suppression, Contrast, and Others) and was trained and tested on 18,181 MRI series from the multicentre PRIMAGE repository. The dataset was split by patient into 80% training/validation and 20% testing; within the training/validation subset, five-fold cross-validation was used. With the exception of contrast classification, all modules used DICOM tag-based machine learning models (CatBoost/Random Forest), while the Contrast classifier incorporated image analysis using a pretrained single-slice ResNet-50. Ethical approval for the study was obtained.

**Results:**

Accuracy was 0.994 for Weighting, 0.984 for Family, 0.959 for Fat suppression and 0.958 for Others; the Contrast classifier recorded 0.841. Overall, the end-to-end classification yielded a weighted F1 of 0.849 (CI: 0.837–0.861) and an accuracy of 0.853 (CI: 0.841–0.865).

**Conclusion:**

The proposed approach provides a reliable and scalable solution for labelling large, heterogeneous MRI datasets across multiple anatomical regions. The pipeline achieved excellent performance for Weighting and Family classification, solid performance for Fat Suppression and ‘Others’. However, Contrast classification remains the main limitation and warrants further refinement and/or additional modules.

**Relevance statement:**

Reliable, automated MRI sequence labelling enables faster, reproducible cohort selection and protocol harmonisation in large archives, supporting downstream clinical research and AI tools while reducing manual curation effort and error.

**Key Points:**

- Multicentre PRIMAGE cohort (18,181 MR series) enabling evaluation in a heterogeneous setting.
- Machine learning model combining DICOM metadata and image features.
- High accuracy in series classification: up to 0.994 on key tasks.
- A scalable pipeline reduces manual annotation workload for radiologists.

**Graphical Abstract:**

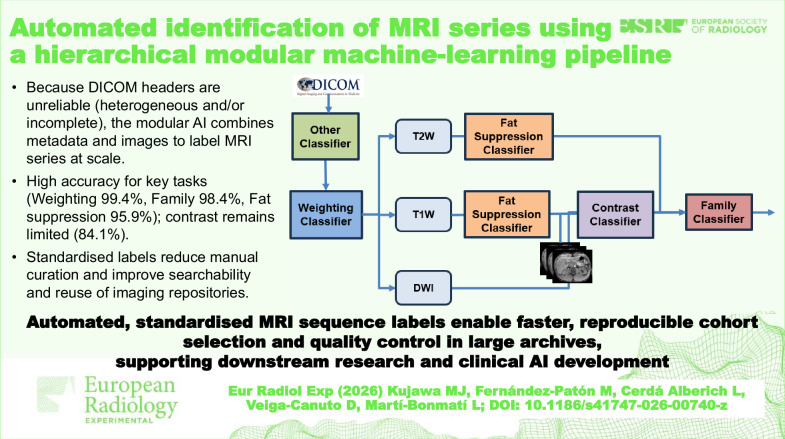

## Background

Due to the rapid growth of imaging data and the increasing demand for annotated datasets for AI development, effective automated methods are urgently needed to label image series in large repositories. Such an annotation process is essential for training and validating AI models (*e.g*., for volumetric segmentation and radiomic biomarker extraction) yet remains challenging in secondary-use MR research because repository data are highly heterogeneous and MR series names are not standardised [1].

Manual labelling of MR series is impractical at repository scale, as it requires the long-term involvement of highly specialised personnel, increasing time and costs and creating a bottleneck in the research workflow, while the repetitive review also increases the risk of human error. These limitations can be mitigated by an effective AI solution for consistent MR series labelling [1, 2]. In some applications, such as dedicated brain MR imaging, existing models analyse series at a high speed and accuracy (0.4 ms per series and 0.9996 accuracy) [3].

AI-based labelling methods may use DICOM metadata, image attributes, or both. DICOM is a widely used standard for storing, transferring, and archiving medical images and associated manufacturer-provided metadata, which include structured tags describing the patient, examination, equipment, and acquisition parameters [4].

In practice, DICOM headers vary across manufacturers, software releases, and centres, and key descriptive fields (*e.g*., “Sequence Name” and “Series Description”) are often incomplete, inconsistent, language-dependent, or removed during anonymisation [1, 2, 5]. These issues make a simple solution based directly on DICOM headers impossible; however, modern tabular machine learning models can be trained to tolerate missingness and vendor variability. We therefore adopt a pragmatic compromise: rather than relying on natural-language descriptors, we leverage a curated set of DICOM-derived features.

Some approaches based on deep learning models that directly analyse the MR images have been proposed, both for 2D and 3D images [6, 7]. These models are not affected by incomplete or incorrect DICOM headers. Previous image analysis-based models have been trained using selected datasets limited to the brain [2, 7, 8], prostate [9, 10], and abdomen [11]. These targeted models exhibit resistance to incomplete or incorrect headings, even in heavily anonymised datasets. Studies covering several anatomical regions, including the chest, abdomen and pelvis, are uncommon. Other studies propose a metadata-based solution, which is more resistant to different anatomical regions, as DICOM metadata does not vary much [6]. Additionally, metadata analysis models offer several advantages, including speed of analysis, low computing power requirements and do not require GPUs [1, 3].

Notably, an integrated approach that combines image-derived features and DICOM metadata within a single model has not been demonstrated in prior work to date. Among the studies we reviewed, Pizarro et al [8] evaluated both paradigms, but as separate models rather than a hybrid fusion strategy: an image-based deep learning pipeline (convolutional neural network on a single slice followed by a dense neural network for volume-level inference) was compared against a Random Forest model trained on metadata-derived features for series identification. Similarly, Pan et al [12] used an image-based deep learning classifier, while metadata from JSON headers served only to guide the initial labelling/categorisation process and were not used as model inputs.

We aimed to improve automatic labelling of MR imaging series in a large, heterogeneous radiological dataset from a multicountry research platform. Building on prior PRIMAGE-related work demonstrating repository-scale labelling from DICOM metadata, we hypothesised that combining DICOM metadata with targeted image analysis would optimise series annotation, particularly for determining contrast administration. Accordingly, we introduce a hierarchical, clinically grounded modular label space (Family/Weighting/Fat Suppression/Contrast/Others) that can be recombined into routine protocol labels, and a hybrid modelling strategy in which modules rely on DICOM tag–based tabular machine learning, while contrast is handled by a pretrained single-slice ResNet-50 classifier.

## Methods

### Ethics

Ethical approval was obtained from the Ethics Committee for Investigation with medicinal products of the University and Polytechnic La Fe Hospital (ethic code: 2018/0228, 27 March 2019). The recommendations of the MAIC-10 framework were considered and applied in the design and reporting of this study [13].

### Study design

**Fig. 1:**
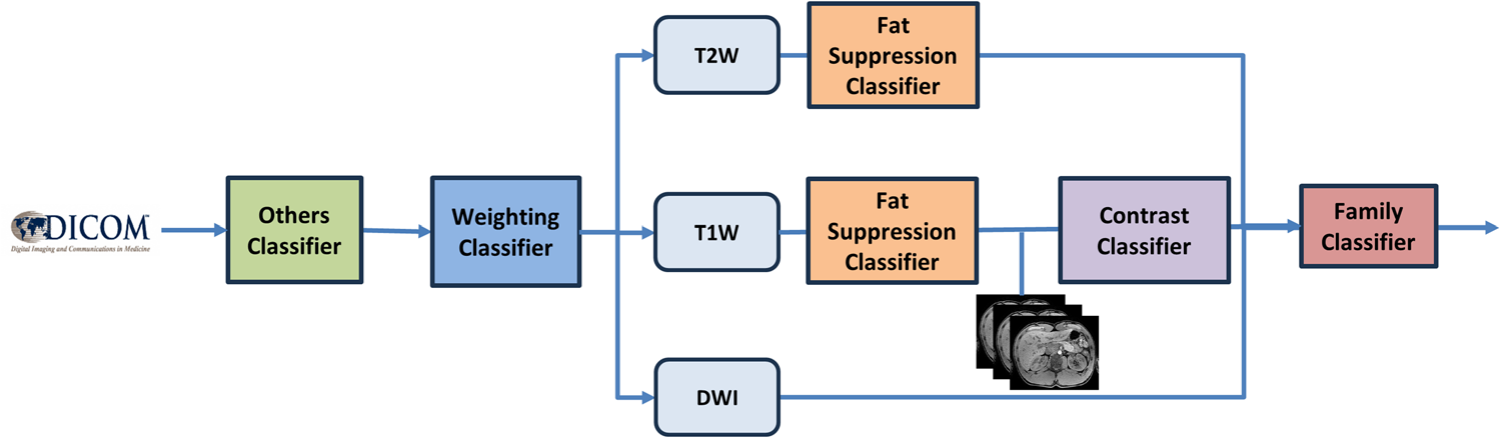
Schematic overview of the study pipeline: extraction of DICOM tags (with representative image information), and sequential classification to generate standardised series labels. Image-based input was used only in the Contrast classifier, which directly analysed T1-weighted (T1W) series

The schematic overview of the study pipeline is shown in Fig. 1.

### Dataset

All MRI series available in the Horizon 2020 PRIMAGE project repository (PRedictive *In silico* Multiscale Analytics to support cancer personalised diagnosis and prognosis, empowered by imaging biomarkers) were included, with no additional inclusion or exclusion criteria applied. PRIMAGE was a retrospective, multicentre, international consortium that collected MRI, CT, and SPECT imaging data, along with clinical, pathological, laboratory, and genetic information from paediatric patients with a neuroblastic tumour [14]. The analysis comprised 18,181 MR series from 1,198 anonymised MR studies performed in 703 patients. Several European centres from Austria, Germany, Spain, Italy, Romania, and Lithuania participated in the study. Age at diagnosis was 34 ± 36 months (mean ± SD; range 0–245 months), with balanced sex distribution (323 females, 380 males). All patients had at least one MR study at diagnosis, and some had follow-up MR imaging.

MR studies covered diverse anatomical regions (brain to lower limbs) and were acquired on 1.5-T and 3-T scanners from multiple vendors: Siemens Healthineers (*n* = 10,734), Philips (*n* = 4,182), General Electric (*n* = 3,162) and Toshiba (*n* = 103). The distribution of MR characteristics is shown in Table 1.

**Table 1:** Number of MR series categorised by class

Category	*n*
Total	18,181
Others	1,310
Series to analyse	16,871
Weighting	
T1W	9,489
T2W	5,832
DWI	1,550
Fat suppression	
Yes	8,249
No	7,072
Contrast	
Yes	7,272
No	2,217
Family	
Spin echo (SE)	7,637
Gradient recalled (GR)	6,647
Inversion recovery (IR)	425
Echo planar (EP)	761

“Others” denotes non-diagnostic data (*e.g*., localisers, respiratory curves) and underrepresented diagnostic series excluded from the main analysis, whereas “Series to analyse” includes diagnostic MR acquisitions

### Classifier architecture

The proposed sequential architecture consisted of five conditionally connected classifiers. The output of each classifier determines whether the next one is activated, as illustrated in Fig. 1. This hierarchical structure enables a progressive and efficient classification of MR images based on specific characteristics. Throughout this article, we refer to the individual classifiers as follows:

- Others (green): serves as the initial filter, aiming to distinguish clinically relevant MR series for further analysis (T1W, T2W, and DWI) from other types of images (such as parametric maps, localisers, calibration scans, and screenshots). In addition, this category encompassed series that, due to their very limited representation in our repository, were not included in the analysis, such as susceptibility-weighted imaging, T2*W, Proton Density, and Perfusion weighted series. If the series is classified as other types of images, its classification stops in this model.
- Weighting (blue): once a relevant series is identified, this model classifies the image according to its weighting type, distinguishing among T1W, T2W, and DWI. This model will only be activated if the *Others model* classifies the series as relevant for further analysis. If the series is classified as DWI, the top branch classification stops in this model.
- Fat Suppression (orange): determines whether a fat suppression technique has been applied during image acquisition. This model will only be activated if the *Weighting model* classifies the series as T1W or T2W.
- Contrast (purple): an image-based classifier that identifies the presence or absence of contrast in the series. This model will only be activated if the *Weighting model* classifies the series as T1W.
- Family (red): the final model determines the echo family to which the series belongs (IR: Inversion Recovery, EP: Echo Planar, GR: Gradient Echo and SE: Spin Echo), if the DICOM Scanning Sequence tag is empty or contains the value Mode Research. This model will only be activated if the *Others model* classifies the series as relevant for further analysis.

With the exception of the contrast classifier, all other single models are based on tabular data extracted from DICOM tags.

### Preliminary classifier and DICOM tags preprocessing

A preliminary text-based classifier was applied to the DICOM tags: Series Description, Sequence Name, and Protocol Name to assign each series to a coarse textual category. This preliminary class was not used as a final prediction, but as an engineered feature. For the downstream classifiers, it was concatenated with the preprocessed DICOM tag features to form a single tabular input. The DICOM-derived variables included selected metadata features extracted from the series. Both the categorical variables and the class derived from the previous model were one-hot encoded to facilitate their use in machine learning models. Detailed information on the preliminary classifier and DICOM tags preprocessing is provided in the Supplementary Material.

### DICOM tag selection

To identify the most relevant features for each classifier, we performed a statistical correlation analysis between the available variables and each model’s target variable, using a significance level of 0.01 for all tests (the full procedure and selected feature lists are provided in the Supplementary Material). The resulting feature sets were used as inputs to the downstream tabular models. The DICOM tags selection was performed only in the train split.

### DICOM image selection for the image-based model

For the contrast classifier, we extracted a single representative 2D image per MR series by selecting the central slice from a representative volume. In series with multiple volumes (*e.g*., multiple b-values or reconstruction types), a predefined selection rule was applied to choose the appropriate volume, after which the central slice was taken to provide a spatially balanced representation. Full selection rules and keyword criteria are provided in the Supplementary Material.

### Annotation of MR series

**Fig. 2:**
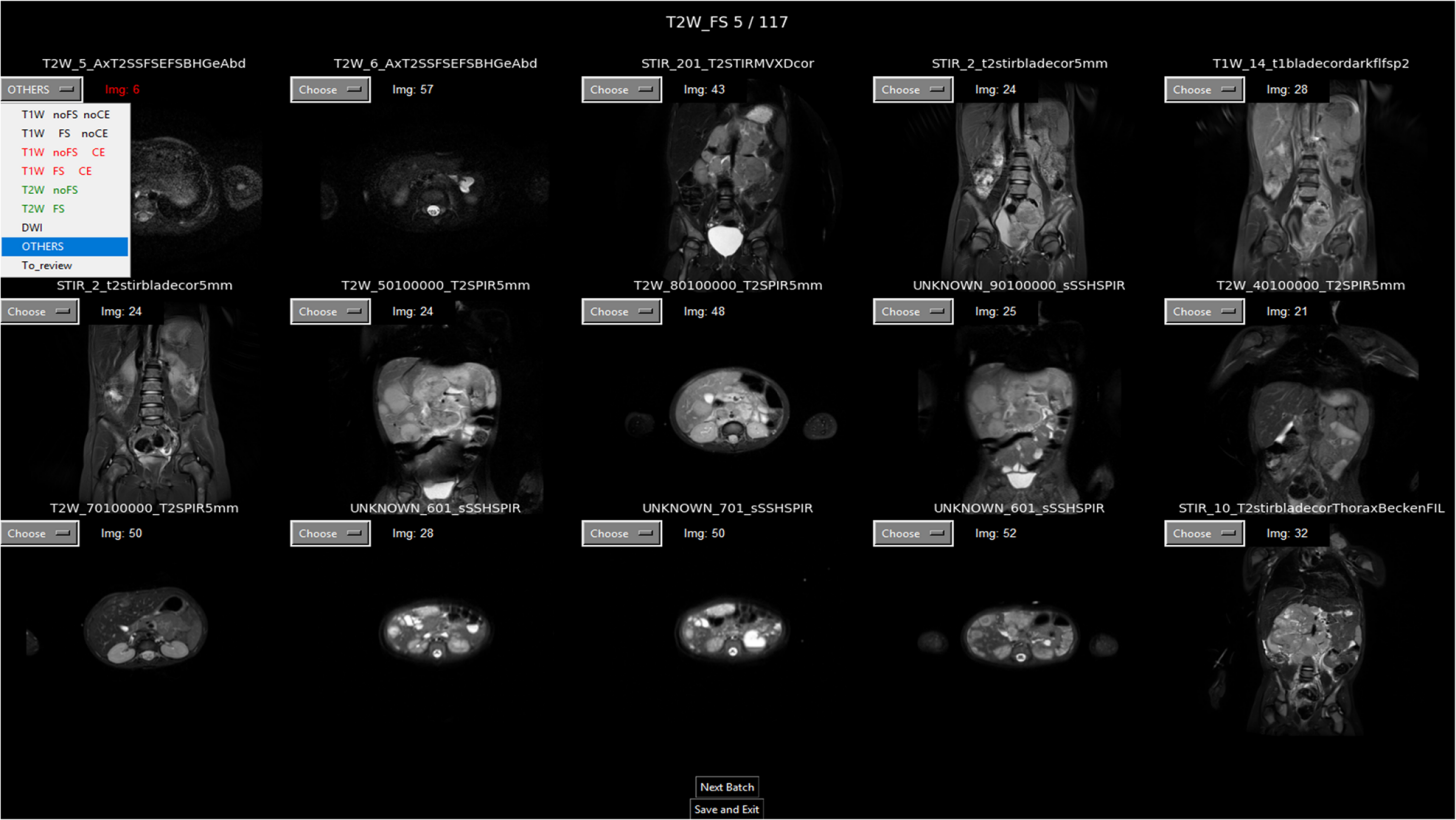
Screenshot of the tool developed for reviewing preliminary classifications

All MR series were annotated by a board-certified radiologist (10 years’ experience). Initially, 6,120 series were manually labelled in a DICOM viewer (RadiAnt DICOM Viewer v2025.1, 64-bit; Medixant) by reviewing all images in each series and assessing characteristic imaging features of the corresponding sequence. Contrast enhancement was determined based on the visible enhancement of tissues and blood vessels. This subset was used to train a preliminary model, which then performed coarse automated classification of the remaining 12,061 series to accelerate expert review. All classifications were subsequently manually verified by a radiologist using a tool dedicated to this study that presents a 5 × 3 matrix of central slices from different series, grouped by the initial annotation (Fig. 2). This layout enables simultaneous assessment of 15 MR series and removes the need to retrieve individual series from folders, load them into the DICOM viewer, and verify patient/series identifiers. The radiologist instead corrected mislabelled series by selecting the appropriate label from predefined categories or marking series as “To review” when uncertain. All “To review” series were subsequently re-opened and re-assessed by full manual inspection of the entire series in the DICOM viewer, after which a definitive final label was assigned; no series were excluded on this basis (*n* = 156; 1.3% of the 12,061 automatically preclassified series). The resulting final annotated dataset was used to train, validate, and test the definitive model.

### Model training

The dataset was divided into 80% for training/validation and 20% for testing. Within the training/validation subset, five-fold cross-validation was applied, with 90% used for training and 10% for validation in each iteration. The datasets were split by patient, with the series balanced in each split. Two DICOM tag–based classifiers were evaluated: CatBoost [15] and Random Forest [16]. Both are tree-based ensemble methods suited to structured data and advantageous for imbalanced classes due to class-weighting support and robust generalisation. CatBoost efficiently handles categorical features with minimal preprocessing, whereas Random Forest is simple, stable, and resistant to overfitting. For Random Forest, we used entropy as the split criterion to measure node purity. Its hyperparameters include a maximum depth of nine levels to prevent overfitting and the use of the logarithm base two to determine the number of features to evaluate at each split, while maintaining the pruning parameter at zero. The CatBoost model was configured using logarithmic loss as the objective function, a depth of six, an L2 regularisation of three, 64 border counts, and a plain boosting scheme.

A pretrained ResNet-50 model was employed for the contrast classification [17]. The last three convolutional blocks were unfrozen and retrained for fine-tuning. Images were resized to 224 × 224 pixels; training used a batch size of 128 for 100 epochs. The model was optimised using the binary cross-entropy loss function, with ReLU activations in the intermediate layers and a sigmoid activation in the final output layer. Training employed Adam optimiser (initial learning rate 1e-5) with a ReduceLROnPlateau scheduler (reduction factor 0.8, patience 10 epochs, minimum learning rate 1e-7) to improve stability and convergence.

## Results

Model performance was assessed using the following evaluation metrics: Accuracy, Area Under the ROC Curve (AUC), F1-score, Precision, and Sensitivity (Recall). All confidence intervals (CIs) reported in this study were calculated at the 95% level. As shown in Table 2, the best overall performance was achieved by the Weighting and Family classifiers, which reached near-perfect accuracy and AUC values on both the validation and test sets. Strong and consistent results were also observed for the Others and Fat Suppression models. The Contrast classifier showed the lowest overall performance, with clearly lower Accuracy and AUC despite relatively high F1-score and Sensitivity, indicating that contrast classification was the most challenging task among the evaluated modules. Validation and test metrics remained generally similar across classifiers, suggesting good stability and limited overfitting.

**Table 2:** Main performance metrics for the classifiers

		Accuracy	AUC	F1-score	Precision	Sensitivity
Others	Validation	0.957 ± 0.010	0.921 ± 0.021	0.973 ± 0.006	0.961 ± 0.011	0.985 ± 0.008
	Test	0.958	0.978	0.958	0.958	0.959
Weighting	Validation	0.994 ± 0.001	0.999 ± 0.001	0.994 ± 0.001	0.994 ± 0.001	0.994 ± 0.001
	Test	0.994	0.999	0.993	0.993	0.993
Fat suppression	Validation	0.959 ± 0.004	0.959 ± 0.003	0.961 ± 0.003	0.964 ± 0.004	0.959 ± 0.003
	Test	0.959	0.987	0.960	0.959	0.960
Contrast	Validation	0.846 ± 0.008	0.838 ± 0.020	0.902 ± 0.019	0.884 ± 0.018	0.919 ± 0.021
	Test	0.841	0.839	0.907	0.883	0.914
Family	Validation	0.983 ± 0.002	0.998 ± 0.001	0.983 ± 0.002	0.983 ± 0.002	0.983 ± 0.002
	Test	0.984	0.999	0.942	0.936	0.949

For the validation set, values are reported as mean ± SD across cross-validation folds

Finally, the values for the complete classification, excluding the family, are shown for the eight possible classes in Table 3.

**Table 3:** Confusion matrix for the complete classification, excluding the family category

Predicted (→)/ Ground truth (↓)	DWI	OTHERS	T1W_nFS_nCE	T1W_nFS_CE	T1W_FS_nCE	T1W_FS_CE	T2W_nFS	T2W_FS
DWI	306	3	0	0	0	0	0	2
OTHERS	67	115	6	17	5	38	8	4
T1W_nFS_nCE	0	0	259	99	4	3	0	0
T1W_nFS_CE	0	0	69	273	6	12	1	0
T1W_FS_nCE	0	0	4	4	28	43	0	0
T1W_FS_CE	0	12	10	23	42	1,004	0	4
T2W_nFS	1	0	0	3	1	0	667	19
T2W_FS	2	0	0	0	0	1	22	452

Labels: the suffix denotes fat suppression (*FS* Fat-suppressed, *nFS* Not fat-suppressed); for T1W labels only, the subsequent suffix denotes contrast status (*CE* Contrast-enhanced, *nCE* Non-contrast). Contrast status is not assessed (N/A) for T2W/DWI and is therefore omitted from their label names

The confusion matrix (Table 3) and the final metrics (Table 4) confirm that the model performs well in distinguishing most MR series types. The highest F1-scores were obtained for the T2W series, both without fat suppression (T2W_nFS, F1 = 0.960) and with fat suppression (T2W_FS, F1 = 0.944), as well as for T1-weighted with both fat suppression and contrast (T1W_FS_CE, F1 = 0.914). The DWI class also showed excellent recall (0.984) and a strong F1-score of 0.891.

**Table 4:** End-to-end classification performance for final MR sequence labels

	Precision (CI)	Sensitivity (CI)	F1-score (CI)	*N*
OTHERS	0.885 (0.823–0.938)	0.442 (0.375–0.502)	0.590 (0.522–0.648)	260
DWI	0.814 (0.773–0.851)	0.984 (0.970–0.996)	0.891 (0.865–0.915)	311
T1W_nFS_nCE	0.744 (0.697–0.791)	0.710 (0.660–0.756)	0.727 (0.687–0.764)	365
T1W_nFS_CE	0.652 (0.599–0.697)	0.756 (0.714–0.801)	0.700 (0.661–0.736)	361
T1W_FS_nCE	0.326 (0.234–0.423)	0.354 (0.259–0.451)	0.339 (0.250–0.421)	79
T1W_FS_CE	0.912 (0.895–0.927)	0.917 (0.901–0.932)	0.914 (0.902–0.926)	1,095
T2W_nFS	0.956 (0.939–0.970)	0.965 (0.951–0.978)	0.960 (0.950–0.970)	691
T2W_FS	0.940 (0.917–0.960)	0.948 (0.925–0.967)	0.944 (0.928–0.958)	477
Macro avg	0.778 (0.761–0.796)	0.760 (0.743–0.778)	0.758 (0.741–0.775)	3,639
Weighted avg	0.858 (0.847–0.870)	0.853 (0.841–0.865)	0.849 (0.837–0.861)	3,639
Accuracy	0.853 (0.841–0.865)			3,639

Values are reported as point estimates with 95% confidence intervals. Macro and weighted averages are provided. Accuracy is reported for the overall dataset. *N* denotes the number of series in each class. Results are reported for the independent test set (20% of the dataset; *N* = 3,639)

By contrast, lower performance was observed in series defined by more than one classification, such as T1W_nFS_CE (F1 = 0.700) and T1W_nFS_nCE (F1 = 0.727), while the T1W_FS_nCE class showed the weakest results overall (F1 = 0.339), suggesting difficulty in distinguishing the contrast. The OTHERS class also showed a moderate precision (0.885), reflecting its heterogeneity.

Overall, the model achieved a macro-averaged F1-score of 0.758 and a weighted average of 0.849, with a global accuracy of 0.853.

To evaluate the robustness and generalisation capacity of the model in the face of clinical heterogeneity, a stratified performance analysis was performed. Table 5 presents the detailed evaluation of metrics divided according to the manufacturer of the equipment, magnetic field strength, and country of origin of the data.

**Table 5:** Subgroup analysis of model performance according to scanner manufacturer, magnetic field strength, and country of origin

Factor	Subgroup	Accuracy (CI)	Sensitivity macro (CI)	Sensitivity weighted (CI)	Precision macro (CI)	Precision weighted (CI)	F1 macro (CI)	F1 weighted (CI)	*N*
Manufacturer	General Electric	0.857 (0.827–0.885)	0.742 (0.696–0.786)	0.857 (0.827–0.885)	0.807 (0.742–0.856)	0.853 (0.823–0.887)	0.758 (0.705–0.802)	0.850 (0.818–0.883)	537
	Philips	0.844 (0.818–0.868)	0.745 (0.695–0.798)	0.844 (0.819–0.869)	0.738 (0.701–0.773)	0.853 (0.828–0.879)	0.734 (0.690–0.772)	0.846 (0.818–0.870)	808
	Siemens	0.855 (0.840–0.869)	0.758 (0.735–0.781)	0.855 (0.841–0.869)	0.772 (0.753–0.794)	0.866 (0.852–0.879)	0.751 (0.728–0.774)	0.851 (0.836–0.866)	2,272
	Toshiba	0.773 (0.591–0.909)	0.569 (0.356–0.625)	0.773 (0.591–0.955)	0.600 (0.344–0.625)	0.782 (0.554–1.000)	0.580 (0.344–0.618)	0.772 (0.548–0.933)	22
Magnetic field strength	1.5 T	0.853 (0.840–0.866)	0.760 (0.744–0.776)	0.853 (0.840–0.866)	0.779 (0.764–0.794)	0.859 (0.846–0.872)	0.759 (0.743–0.775)	0.850 (0.837–0.863)	2,745
	3 T	0.850 (0.827–0.873)	0.757 (0.729–0.785)	0.850 (0.827–0.873)	0.776 (0.749–0.803)	0.856 (0.833–0.879)	0.756 (0.728–0.784)	0.848 (0.825–0.871)	894
Country	Austria	0.854 (0.832–0.876)	0.761 (0.734–0.788)	0.854 (0.832–0.876)	0.780 (0.754–0.806)	0.860 (0.838–0.882)	0.760 (0.733–0.787)	0.851 (0.828–0.874)	958
	Spain	0.853 (0.827–0.879)	0.760 (0.728–0.792)	0.853 (0.827–0.879)	0.779 (0.748–0.810)	0.859 (0.833–0.885)	0.759 (0.727–0.791)	0.850 (0.824–0.876)	706
	Italy	0.900 (0.800–1.000)	0.818 (0.558–0.875)	0.900 (0.767–1.000)	0.787 (0.600–0.875)	0.957 (0.918–1.000)	0.787 (0.576–0.868)	0.917 (0.804–1.000)	30
	Lithuania	0.825 (0.699–0.925)	0.597 (0.452–0.700)	0.825 (0.700–0.925)	0.600 (0.458–0.700)	0.813 (0.665–0.958)	0.597 (0.445–0.682)	0.818 (0.676–0.936)	40
	Romania	0.905 (0.762–1.000)	0.562 (0.375–0.625)	0.905 (0.762–1.000)	0.551 (0.348–0.625)	0.861 (0.678–1.000)	0.557 (0.359–0.625)	0.882 (0.718–1.000)	21
	Germany	0.851 (0.835–0.867)	0.758 (0.739–0.777)	0.851 (0.835–0.867)	0.777 (0.758–0.796)	0.857 (0.841–0.873)	0.757 (0.738–0.776)	0.848 (0.832–0.864)	1,884

Values are reported as point estimates with 95% confidence intervals. Macro and weighted metrics are shown. *N* denotes the number of series in each subgroup

Overall, the model demonstrated stable performance. When analysing manufacturers, the metrics remained stable among the predominant brands (General Electric, Philips, and Siemens), showing an accuracy that ranged closely between 0.844 and 0.857. The only exception was observed in studies performed with Toshiba equipment, which presented significantly lower values (Accuracy of 0.773 and F1 Macro of 0.580). Regarding magnetic field strength, performance was almost identical between 1.5 T and 3 T.

In terms of distribution by country, the model remained consistent in the countries with the highest representation in the dataset, such as Germany, Austria, and Spain. However, atypically higher metrics were recorded in the cohorts from Italy (Accuracy of 0.900) and Romania (Accuracy of 0.905). It is essential to note that the divergences observed both in the Toshiba manufacturer and in the countries of Italy and Romania correspond to the subgroups with the lowest number of cases (*N* = 22, *N* = 30, and *N* = 21, respectively).

## Discussion

This study uses the PRIMAGE repository, a unique collection of MR scans from neuroblastoma patients, which provides multiple anatomical regions, multi-plane acquisitions across several vendors and countries [14]. Such diversity is important for developing AI models that generalise to heterogeneous data. This distinguishes our work from most previous studies, except those by Kim et al [18] and Gomis et al [6], which typically used single-region repositories (most often brain or prostate). Kim et al [18] analysed thorax, abdomen, and pelvis data, and Gomis et al [6] likewise used PRIMAGE.

Existing AI models typically distinguish only between basic MR series. Most commonly, they have focused on T1W, T1W CE, T2W, and FLAIR [2, 7, 8], while a few studies have also analysed other series such as DWI, susceptibility-weighted imaging, or DCE [1, 3, 6, 18]. In our study, a modular approach was used to create a set of AI models whose output was defined according to the physical and technical principles of MR series acquisition (family, weighting, fat suppression, and contrast enhancement), which can be combined to represent ≥ 18 composite series labels encountered in routine practice [19, 20]. An additional advantage is that the modular design may facilitate future expansion and adaptation to evolving scanner technologies and user needs, as individual stages could potentially be added or modified without necessarily retraining the entire system, which may in turn support longer-term robustness.

In recent years, methods for the automatic classification of MR series have advanced rapidly using both image analysis and DICOM metadata. Image-driven deep learning approaches have reported excellent performance in constrained settings, for example, achieving accuracies of 0.992 in brain series classification using either multiple 2D images (Ranjbar et al [7]) or a single mid-slice (Pan et al [12]). Nevertheless, generalisation across domains can remain challenging; Kim et al [18] reported an overall accuracy of 0.994, but performance dropped substantially under cross-vendor evaluation (F1-score 0.865). In parallel, metadata-based methods relying on DICOM tags have also achieved excellent performance, with Random Forest models reporting overall accuracy consistently exceeding 0.999 in large brain MRI cohorts (Liang et al [1]) and accuracies ranging from 0.974 to 0.9996 across two datasets (Gauriau et al [3]), while CatBoost-based approaches have shown more moderate performance in highly heterogeneous multicentre settings (combined accuracy 0.88) [6]. Against this background, our results for Weighting, Family, Others, and Fat Suppression are comparable to prior work despite the greater heterogeneity of the input data and broader label space (Table 2). However, the Contrast classifier remained the limiting component, with validation metrics of 0.838–0.919 and lower test performance (Accuracy 0.841, AUC 0.839), although its F1-score (0.907) and sensitivity (0.914) were relatively strong.

The issue of imprecise discrimination between contrast-enhanced and non-contrast series has been reported in several previous studies, including the works of Pizarro et al [8], Mello et al [2], and Gauriau et al [3]. This limitation may arise from the fact that the DICOM tag “Contrast/Bolus Agent (0018,0010)” is often not completed, while other acquisition parameters in T1-weighted nCE and CE series can be identical, thereby reducing the utility of metadata for differentiation. In addition, label noise may arise because contrast administration is inconsistently documented, and the reference label may sometimes reflect the intended protocol rather than unequivocal enhancement visible in the images. An important factor that may have influenced the results in the Contrast model also appears to be variability in post-contrast enhancement patterns across different anatomical regions, and a central slice may not intersect vessels or enhancing structures. Timing and phase variability introduce additional complexity, as post-contrast series may be acquired at different phases (arterial, portal venous, delayed), each exhibiting distinct enhancement characteristics that challenge classification.

When these modular predictions are combined to evaluate the final labels, a natural drop in overall metrics is observed. The discrepancy between the high accuracy of the modules and the moderate end-to-end performance is mainly due to the error propagation inherent in conditional pipelines. Even with minimal error rates per module, the system’s overall reliability follows the product of individual probabilities, resulting in cumulative degradation as the chain lengthens. Therefore, failures in the early stages cause the algorithm to either stop or continue along incorrect paths, generating inaccurate results. This occurs even when each module is trained using ‘clean’ reference data rather than the results of its predecessors. This creates a chain reaction whereby deviations in the early stages lead to significant failures in the later stages.

Beyond this cumulative error, the end-to-end system is also heavily influenced by dataset distribution. The results demonstrate a clear correlation between class support and predictive performance. Majority classes such as T1W_FS_CE (*N* = 1,095), T2W_nFS (*N* = 691), and T2W_FS (*N* = 477) achieved excellent end-to-end F1-scores of 0.914, 0.960, and 0.944, respectively. In contrast, the model’s performance significantly degrades in extreme minority classes, most notably in T1W_FS_nCE (*N* = 79), which obtained an F1-score of only 0.339. This degradation explains the notable gap between our Weighted F1 (0.849), which is dominated by the well-represented classes and the Macro F1 (0.758), which heavily penalises the model for its struggles with rare series. Furthermore, lower performance is not solely dictated by sample size but also by class heterogeneity; for instance, the OTHERS class (*N* = 260) yielded a moderate F1-score of 0.590 due to its intrinsically diverse nature as a “catch-all” category. Conversely, the DWI class maintained a high F1-score (0.891) despite a moderate prevalence (*N* = 311), suggesting that when sequence features are highly distinct, the model is less reliant on massive amounts of data to achieve robust classification.

The model’s generalisation ability was also evaluated in light of the inherent variability in multicentre clinical data. As detailed in Table 5, overall performance proved to be highly consistent across different scanners, magnetic field strengths, and geographic regions, thus responding to the critical need for robust tools for real-world clinical practice. The model maintained a remarkably stable level of accuracy and F1-score among the leading manufacturers in the cohort (Siemens, Philips, and General Electric), as well as between 1.5-T and 3-T configurations and countries. Despite this, a decrease in overall performance was observed for the manufacturer Toshiba, in contrast to atypically high metrics for the cohorts from Italy and Romania. These three subgroups constitute the extreme minority classes in our study, with very small sample sizes for a robust population assessment (*N* = 22 for Toshiba, *N* = 30 for Italy, and *N* = 21 for Romania).

The limitations discussed above should be noted, along with several additional aspects. First, the series designated for testing were randomly selected from the same repository as those used for training; as emphasised by Matta et al [21], the lack of domain separation across independent centres and scanner vendors limits the ability to reliably assess true out-of-cohort generalisation. Second, image-based inference in the Contrast classifier relied on a representative mid-slice rather than full volumetric information, which may reduce sensitivity to subtle or spatially heterogeneous enhancement. Third, all annotations and final label verification were performed by a single radiologist; therefore, inter-reader agreement was not assessed, and residual label uncertainty—particularly for contrast enhancement—cannot be excluded.

Future development efforts should prioritise improving the Contrast Classifier, as this remains the primary limitation. One potential improvement could involve incorporating the entire image volume into the analysis rather than relying solely on a single mid-slice. However, such an approach would require substantially greater computational resources and would significantly increase processing time [3, 6]. Incorporating an additional module for automatic anatomical region recognition, as described by Tóth et al [22], could improve accuracy further by pre-assigning slices to specific body regions. In the longer term, linking the validation interface directly to the classifier could enable adaptive learning from user corrections. This enables the efficient review and correction of misclassified T1W CE and T1W nCE series by presenting multiple series grouped by their preliminary labels. Another key area for future work is external validation to quantify how well the proposed pipeline generalises beyond the current PRIMAGE cohort. This should involve applying the full labelling workflow to independent, heterogeneous multicentre datasets, including studies acquired at other institutions and on different scanner vendors, to assess robustness and support interoperability with existing repositories.

Alongside advances in the development of automatic series labelling tools, efforts should be made to introduce a unified standard for MR series nomenclature that would be widely adopted. This would facilitate the integration of imaging repositories with other biorepositories [23] and accelerate the development of personalised medicine based on the analysis of complex sets of biomarkers. However, it should be noted that even after the implementation of such a standard, a vast amount of data already stored in existing research and hospital repositories will remain, and its effective use will require tools such as the model developed in our study.

This study presents a modular AI-based framework for automated MR series classification across multiple anatomical regions, providing an efficient and reliable solution for labelling heterogeneous imaging data. The model demonstrated excellent results for the Weighting and Family classifiers, and solid performance for Fat Suppression categorisation. While image-derived features added limited value overall, they slightly improved contrast classification. However, the Contrast classifier remained challenging, underscoring the complexity of distinguishing enhanced from non-enhanced series. These findings establish a solid basis for future developments in automated MR labelling and AI-assisted medical imaging workflows.

## Supplementary information

**Additional file 1: Table S1.** Summary of variables extracted from DICOM tags.

## Data Availability

The datasets analysed during the current study are not publicly available due to institutional restrictions and patient confidentiality policies. However, de-identified data may be shared upon reasonable request to the corresponding author, subject to institutional approval and applicable ethical and legal requirements.

## Abbreviations

AI: Artificial intelligence
AUC: Area under the ROC curve
CE/nCE: Contrast-enhanced/non-contrast-enhanced
DICOM: Digital Imaging and Communications in Medicine
DWI: Diffusion-weighted imaging
F1: F1-score
FS/nFS: Fat-suppressed/non-fat-suppressed
MRI: Magnetic resonance imaging
Sequence (MRI sequence): An acquisition scheme (RF pulses, gradients, and readout) that defines the contrast mechanism and the imaging family
Series (MRI series): A coherent set of images representing one executed instance of a sequence with specific parameters, geometry, and reconstruction, stored and viewed as a single unit within an examination
Study: A single imaging examination session, typically comprising multiple series/sequences acquired under one protocol
T1W: T1-weighted
T2W: T2-weighted
