# Supplementary material for: Automated identification of MRI series using a hierarchical modular machine-learning pipeline
Source: Eur Radiol Exp. 2026 May 28;10:77. doi: 10.1186/s41747-026-00740-z (PMC13219728; doi:10.1186/s41747-026-00740-z)
Supplement: Supplementary file 1 — Additional file 1: Table S1. Summary of variables extracted from DICOM tags. [file 41747_2026_740_MOESM1_ESM.pdf]

# Automated identification of MRI series using a hierarchical modular machine-learning pipeline

## ELECTRONIC SUPPLEMENTARY MATERIAL

Preliminary classifier

The series were initially checked using a preliminary classifier based on pattern recognition applied to textual information found in the DICOM tags Series Description, Sequence Name, and Protocol Name. This classifier performs automatic categorization of the sequences based on the content of these fields. The output classes defined for this model include the following categories: PROCESSED, IVIM, DW, PW, STIR, ChS, SCREENSAVE, CALIBRATION, LOCALIZER, FLAIR, T1W, T2W, PDW, SWAN, mDIXON, MTONOFF, DTI, rsfMRI, QSM, MECSE and THRIVE.

DICOM tag preprocessing

A preliminary selection of variables was made based on prior scientific literature [6]. To this initial set, the class assigned by the model described in the previous section was added, along with new variables calculated from various DICOM tags. These additional variables include: the number of b-values present in the sequence, the maximum b-value, the total number of images, the number of acquired volumes, Out-phase if the image type contains "OUT_PHASE", "OUTPHASE", In-phase if it contains 'IP', 'INPHASE' or 'IN_PHASE'; Water if it contains 'WATER' or 'W'; or Fat if it contains 'FAT' or 'F'.

Table S1 presents a summary of all the variables considered, organized into three categories: continuous, categorical, and calculated.

Table S1. Summary of variables extracted from DICOM tags.

| Continuous variables | Categorical variables | Calculated variables |
|---|---|---|
| EchoTime | SequenceVariant | Number of b-values |
| InversionTime | ScanningSequence | Maximum b-value |

| | | |
|---|---|---|
| EchoTrainLength | ScanOptions | Total number of images |
| RepetitionTime | ImageType | Number of volumes |
| SliceThickness | | In Phase |
| FlipAngle | | Out Phase |
| PixelBandwidth | | Water |
| EchoNumbers | | Fat |
| PixelSpacing | | |

Additionally, both the categorical variables and the class derived from the previous model were one-hot encoded to facilitate their use in machine learning models. The categories considered for each categorical variable are as follows:

- SequenceVariant: OSP, SS, MP, SP, MTC, SK
- ScanningSequence: IR, EP, GR, SE, MR
- ScanOptions: PER, PFP, FS, PFF, PPG, SP, CG, RG, FC
- ImageType: Primary/Secondary, Original/Derived, Water, Fat, In phase, Out phase

DICOM tag selection

To identify the most relevant features for each classifier, we performed a statistical correlation analysis between the available variables and each model's target variable, using a significance level of 0.01 for all tests. For categorical variables, dependence on classifier output classes was assessed with the Chi-square test.

For continuous variables in the Others, Fat Suppression, and Contrast classifiers, Shapiro–Wilk was used to test normality. If normality was confirmed, Levene's test was used to assess variance homogeneity; then Student's t-test was applied under homoscedasticity, otherwise Welch's t-test. If normality was not met, the non-parametric Mann–Whitney U test was used.

For the Weighting and Family classifiers, we followed a similar approach: after Shapiro–Wilk and Levene's test, we used classical ANOVA under homoscedasticity or Welch's ANOVA under heteroscedasticity; if normality was not satisfied, we applied Kruskal–Wallis. This analysis enabled selection of the most discriminative features for each classifier, improving subsequent model performance.

The selected features for each classifier are as follows:

- Others: PixelSpacing, SliceThickness, MRAcquisitionType, FlipAngle, PixelBandwidth, b_value_num, Num_img,Num_vol, T2W, PROCESSED, UNKNOWN, PW, STIR, T1W, LOCALIZER, mDIXON, DW, FLAIR, PDW, SWAN, ChS, SequenceVariant_OSP, SequenceVariant_SP, SequenceVariant_MP, ScanningSequence_SE, ScanningSequence_EP, ScanningSequence_GR, ScanningSequence_IR, ScanOptions_FS,ScanOptions_PFP, ScanOptions_FC,ScanOptions_PFF, ScanOptions_SP, W , F, IP, OP.
- Weighting: PixelSpacing, RepetitionTime, EchoTime, EchoTrainLength, SliceThickness, MRAcquisitionType, FlipAngle, PixelBandwidth, b_value_num, Num_img and Num_vol.
- Fat Suppression: EchoTime, RepetitionTime, SliceThickness, FlipAngle, PixelBandwidth, ScanningSequence_IR, ScanOptions_FS, Water, Fat, In_Phase, Out_Phase.
- Family: EchoTime, RepetitionTime, FlipAngle, PixelBandwidth.

The DICOM tag selection was performed only in the train split. Missing values for variables PixelBandwidth and EchoTrainLength were imputed with the mean, and MRAcquisitionType with mode of the training dataset.

## DICOM Image Selection for the Image-Based Model

For the contrast classifier, a single representative 2D DICOM image was selected from each series. The process began by estimating the number of volumes, determined based on the repetition of spatial positions within the series. In series with multiple b-values, the volumes were sorted according to the b-value, and the first volume was selected. For series containing different reconstruction types, a priority criterion was applied to select the volume in the following order: in-phase, water suppression, out-

of-phase, and fat suppression. This prioritization was based on the analysis of the DICOM Image Type field, identifying terms such as "IP", "INPHASE", "IN_PHASE", "WATER", "W", "OUT_PHASE", "OUTPHASE", "OP", "OPP_PHASE", "FAT", and "F". For all other series, volumes were sorted based on the Acquisition Time, and the central volume was selected. Finally, from the selected volume, the central slice was extracted according to the acquisition plane, to obtain a spatially balanced representation of the series.
